# Microbial Profile and Antimicrobial Resistance Patterns in Chronic Lower Limb Ulcers: Evidence from a Brazilian Dermatology Referral Center

**DOI:** 10.3390/microorganisms14061199

**Published:** 2026-05-26

**Authors:** Silas Matheus Brosco de Toledo Piza, Regina Maldonado Poz Bernardo, Claudia Alessandra de Lima Ramos, Maria de Lourdes Ribeiro de Souza da Cunha, Patricia Sammarco Rosa, Antônio Carlos Ceribelli Martelli, Luiza Pinheiro-Hubinger-Stauffer

**Affiliations:** 1 Microbiology Laboratory, Instituto Lauro de Souza Lima (ILSL), Bauru 17034-971, SP, Brazil; silas.piza@unesp.br (S.M.B.d.T.P.); prosa@ilsl.br (P.S.R.); 2 Molecular Biology Laboratory, Instituto Lauro de Souza Lima (ILSL), Bauru 17034-971, SP, Brazil; 3 Department of Tropical Diseases, Botucatu Medical School, São Paulo State University (UNESP), Botucatu 18618-689, SP, Brazil; mlrs.cunha@unesp.br; 4 Wound Clinic, Instituto Lauro de Souza Lima (ILSL), Bauru 17034-971, SP, Brazil; regina.bernardo@ilsl.br (R.M.P.B.); claudia.ramos@ilsl.br (C.A.d.L.R.); martelli1@terra.com.br (A.C.C.M.); 5 Electron Microscopy Center, Institute of Biosciences of Botucatu, São Paulo State University (UNESP), Botucatu 18618-689, SP, Brazil

**Keywords:** chronic ulcers, *Pseudomonas aeruginosa*, *Proteus mirabilis*, *Staphylococcus aureus*, antimicrobial susceptibility, β-lactamases

## Abstract

Chronic ulcers are characterized by impaired tissue repair and frequently harbor antimicrobial-resistant microorganisms, worsening clinical outcomes. The objective of this study is to identify microbial agents in chronic ulcers treated at the Lauro de Souza Lima Institute Wound Care Outpatient Clinic and to evaluate their antimicrobial susceptibility profiles and β-lactamase production. Samples (swab and biopsy) from patients treated at the Lauro de Souza Lima Institute were analyzed. Susceptibility was assessed by disk diffusion. ESBL and AmpC production were confirmed by PCR targeting *blaTEM*, *blaSHV*, *blaCTX-M1*, and *blaCMY-2*. In *Staphylococcus* spp., oxacillin and clindamycin resistance were evaluated and confirmed by *mecA* and *ermAC*. From 33 patients (mean age 63.4 years), 116 isolates were obtained, mainly *Pseudomonas aeruginosa* (27%), *Proteus mirabilis* (18%), and *Staphylococcus aureus* (13%). *P. aeruginosa* showed high resistance, with 48% MDR and 29% PDR. Among Enterobacterales, 19% were ESBL producers and 17% AmpC, with 56% carrying *blaCMY-2*. In *Staphylococcus*, 33% were oxacillin-resistant and 50% expressed MLSb phenotype. *P. aeruginosa* was identified as the most prevalent pathogen, with frequent MDR/PDR phenotypes. Resistance genes exhibited a discrepancy between genotypic and phenotypic profiles, suggesting the presence of unexpressed resistance that may be inducible during treatment.

## 1. Introduction

The skin is a highly specialized organ, essential for maintaining homeostasis, acting as a physical, chemical, and immunological barrier [1]. The disruption of this barrier may lead to the formation of ulcers that impair the healing process. In many cases, these lesions fail to progress properly through the stages of tissue repair, remaining in a state of persistent inflammation. Chronic wounds are characterized by delayed or incomplete healing, resulting from host-related factors, vascular alterations, and the continuous presence of microorganisms, which significantly impact quality of life and increase the risk of complications [2,3].

Chronic lower limb ulcers represent a major clinical and public health challenge, particularly among individuals with comorbidities. These lesions are frequently colonized by a complex microbiota where Staphylococcus aureus is identified as the most prevalent pathogen, while Pseudomonas aeruginosa is commonly recovered from deeper tissue layers, often associated with biofilm formation. Both pathogens, along with members of the Enterobacterales order, significantly complicate the healing process due to their intrinsic and acquired resistance mechanisms, which include enzymatic inactivation, target modification, and reduced drug permeability [4]. Consequently, these infections are difficult to treat and are often associated with multidrug resistance, contributing to therapeutic failure, prolonged treatment, and increased healthcare costs. This scenario is part of a critical global trend, as antimicrobial resistance (AMR) is projected to cause 10 million deaths annually by 2050 if effective interventions are not implemented. Therefore, the molecular characterization of resistance genes in chronic wounds is essential to mitigate this burden [5].

Expression of the *mecA* gene confers resistance in *Staphylococcus aureus* to penicillins and cephalosporins, giving rise to the acronym MRSA (*Methicillin-Resistant Staphylococcus aureus*). Similarly, resistance to macrolides, type B streptogramins, and lincosamides defines the MLSb phenotype, encoded by the *erm* gene family (*ermA* and *ermC*). This resistance can be inducible (iMLSb), triggered by exposure to certain antimicrobials, or constitutive (cMLSb) when the resistance is continuously expressed in the microorganism. Among Gram-negative bacteria, extended-spectrum β-lactamases (ESBLs) and class C cephalosporinases (AmpC) act as bacterial enzymes that hydrolyze β-lactam antibiotics. ESBLs confer resistance to all β-lactams except carbapenems and are often associated with the presence of the *blaCTX*, *blaTEM*, and *blaSHV* genes. Meanwhile, AmpC enzymes display similar activity but retain susceptibility to fourth-generation cephalosporins, such as cefepime, and can be detected through the presence of the *blaCMY* gene [6,7].

This study aims to identify the most prevalent bacterial species in chronic lower limb ulcers of patients with chronic diseases, to characterize the antimicrobial susceptibility profile of the isolates, and to detect both the phenotypic and molecular expression of genes involved in antimicrobial resistance.

## 2. Materials and Methods

### 2.1. Sample Collection

A total of 33 samples of exudate and biopsy tissue were collected from chronic lower limb lesions in patients with chronic diseases treated at the Wound Care Outpatient Clinic of the Lauro de Souza Lima Institute (Bauru, São Paulo, Brazil) between September 2022 and July 2024. Ulcer exudates were collected using swabs containing Stuart transport medium, following the technique described by Levine et al. [8]. Subsequently, the lesion was disinfected with 0.5% chlorhexidine, and local anesthesia was administered with lidocaine hydrochloride (20 mg/mL) (Hypofarma, Ribeirão das Neves, MG, Brazil) to perform a deep ulcer biopsy using a sterile 6 mm punch.

Inclusion and Exclusion Criteria: Patients were eligible for the study if they met the following inclusion criteria: (i) presenting with chronic lower limb ulcers (persisting for 4 weeks); (ii) having associated chronic diseases (such as leprosy, diabetes, or hypertension); and (iii) showing failure to respond to conventional wound care treatments. The exclusion criterion was the use of systemic antimicrobial therapy within a minimum 7-day washout period prior to sample collection to ensure the integrity of the microbiological culture results.

Clinical Assessment and Definitions: In this study, the distinction between colonization and infection was not a primary objective, as all lesions were characterized by their chronicity and resistance to standard treatments. During sample collection, a clinical assessment was performed to document the presence of inflammatory signs (erythema, local heat, edema) and ulcer bed characteristics (exudate, slough, and granulation tissue). These clinical features provide context for the microbial landscape found in these wounds, but the study encompasses the total colonizing microbiota of the lesions rather than focusing exclusively on acute infectious processes.

Sample Size Justification: The sample size (*n* = 33) was determined by convenience, encompassing all patients who met the rigorous inclusion criteria at our reference center during the study period. Although the cohort size is limited, it provides a high-quality, detailed microbiological profile of complex, recalcitrant chronic ulcers in a specialized dermatological setting. Consequently, this study serves as a robust exploratory analysis of the local microbial landscape and antimicrobial resistance patterns.

### 2.2. Microbial Culture and Identification

Swab samples were inoculated onto sheep blood agar, MacConkey agar, mannitol agar, and cetrimide agar, and incubated overnight at 37 °C in a bacteriological incubator for the detection of aerobic and facultative anaerobic bacteria. Biopsy samples intended for microbiological culture were processed in sterile saline, inoculated into Brain Heart Infusion (BHI) broth, and incubated overnight at 37 °C. After 24 h, BHI broths showing turbidity were subcultured into the same media used for the swab samples and incubated for an additional 24 h at 37 °C. Bacterial isolates grown on culture media were identified according to the Manual for the Detection and Identification of Bacteria of Medical Importance proposed by the Brazilian Health Regulatory Agency (ANVISA) [9] and by the Bactray identification kit (Laborclin, Pinhais, PR, Brazil), following the manufacturer’s instructions. The accuracy of the biochemical and molecular identification of *Staphylococcus* spp. and Gram-negative bacilli was validated using the following reference strains: *S. capitidis* ATCC 49325, *S. hominis* ATCC 700237, *S. lugdunensis* ATCC 700328, *S. epidermidis* ATCC 35983, *P. aeruginosa* ATCC 27853, and *E. faecalis* ATCC 29212.

### 2.3. DNA Bacterial Extraction

Bacterial DNA extraction was performed according to the procedures recommended in the *Wizard Genomic DNA Purification Kit* manual (FB022, Promega, Madison, WI, USA). The extracted DNA was quantified using a NanoDrop 2000 spectrophotometer (Thermo Fisher Scientific, Waltham, MA, USA) and stored at −20 °C.

### 2.4. Identification of Bacteria of the Genus Staphylococcus

Isolates identified as CoNS (coagulase-negative *Staphylococcus*) were genotypically confirmed by internal transcribed spacer polymerase chain reaction (ITS-PCR), following the protocol proposed by Couto et al. [10]. CoNS isolates that were not identified by ITS-PCR, as well as samples of *Streptococcus* spp. and *Enterococcus* spp., underwent amplification and sequencing according to the protocols described by Pinheiro-Hubinger et al. [11] and Kosecka-Stronjek et al. [12].

### 2.5. Susceptibility to Antimicrobials

Each isolated microorganism was subjected to antimicrobial susceptibility testing using the disk diffusion method, following the technical recommendations, species-specific antimicrobials, and breakpoint criteria provided in the Performance Standards for Antimicrobial Susceptibility Testing issued by the Clinical and Laboratory Standards Institute (CLSI) [13]. Vancomycin susceptibility was assessed using the Etest method (bioMérieux, Marcy-l’Étoile, France) to determine the minimum inhibitory concentration (MIC).

*S. aureus* and CoNS isolates were tested for oxacillin susceptibility using the disk diffusion method with a cefoxitin (30 µg) disk (Oxoid, Basingstoke, UK). To detect *Staphylococcus* spp. strains resistant to clindamycin, the D-test method was applied. A clindamycin disk was placed 20 mm apart from an erythromycin disk. After incubation, the presence of a “D”-shaped flattening of the clindamycin inhibition zone indicated the iMLSb resistance phenotype, whereas the absence of an inhibition halo indicated the cMLSb phenotype [14]. The isolates were also classified as multidrug-resistant (MDR), extensively drug-resistant (XDR), and pan-drug-resistant (PDR). According to the criteria established by Magiorakos et al. [15], MDR bacteria exhibit resistance to three or more antimicrobial classes, XDR strains remain susceptible to no more than two classes, and PDR strains are resistant to all classes tested [15].

### 2.6. Phenotypic Detection of β-Lactamases in Gram-Negative Bacteria

To detect phenotypic ESBL production in Enterobacterales, the disk approximation method was employed using amoxicillin + clavulanic acid disks (20 µg + 10 µg) placed near cephalosporin disks to verify the presence of a “ghost zone,” as described by Giriyapur et al. [16]. The phenotypic disk approximation technique was also used to confirm AmpC β-lactamase production in Enterobacterales and *Pseudomonas aeruginosa*, by placing a ceftazidime disk 20 mm apart from an imipenem (10 µg) disk. The presence of a “D”-shaped flattening of the inhibition zone around either disk indicated AmpC-producing isolates [17].

### 2.7. Resistance Gene Screening by PCR

The presence of the *mecA* gene, associated with oxacillin resistance in *Staphylococcus* spp., was detected by polymerase chain reaction (PCR) following the protocol described by Murakami et al. [18]. Detection of genes involved in MLSb-type resistance (*ermA* and *ermC*) was performed according to the protocols proposed by Moosavian et al. [19] and Lina et al. [20]. Bacteria belonging to the order Enterobacterales were analyzed for genes associated with ESBL (*blaTEM-1*, *blaSHV*, and *blaCTX-M1*) and AmpC (*CMY-2*) production using PCR. For the *blaTEM-1* and *blaSHV* genes, the protocol and primer sequences described by Lee and Yeh [21] and *blaCTX-M1* detection followed that of Thuengern et al. [22]. For the active detection of genes involved in AmpC expression, the *blaCMY-2* gene was identified according to the protocol proposed by Gray et al. [23].

### 2.8. Statistical Analysis

All variable results were analyzed using GraphPad Prism software, version 7.0 (GraphPad Software, San Diego, CA, USA). The Pearson’s Chi-square test and Fisher’s Exact test were applied, and results were considered statistically significant when *p* < 0.05.

## 3. Results

Samples were collected from 33 patients between the second half of 2022 and July 2024. The main sociodemographic characteristics, including gender, age, and educational level, are presented in Table 1. The study population had a mean age of 63.8 years, with a predominance of male participants (64%) and individuals with incomplete education (61%). Most ulcers were of venous origin (91%), of which 58% had been chronic for at least 10 years. Sixty-seven percent of patients reported daily dressing changes, and the most common comorbidity was hypertension, affecting 55% of the patients.

A total of 116 bacterial strains were isolated from chronic lower limb ulcers in 33 patients. A higher prevalence of Gram-negative bacteria (*n* = 77.6%) was observed compared to Gram-positive bacteria (*n* = 39.3%). Only one patient (3%) showed a negative swab culture, while four (12%) had negative biopsy cultures. *Pseudomonas aeruginosa* was the most prevalent species, with 31 isolates (27%), followed by *Proteus mirabilis* (*n* = 21. 1%) and *Staphylococcus aureus* (*n* = 15.1%). Although CoNS species were analyzed separately, this group comprised a total of 15 isolates (13%), ranking among the most prevalent. Similarly, the genus *Staphylococcus* spp. was the most frequent among Gram-positive bacteria (77%) and the second most prevalent overall (*n* = 30), second only to the genus *Pseudomonas* spp. (*n* = 31). The complete list of identified microorganisms is summarized in Table 2.

Among the resistance profiles of the isolates, Gram-negative bacteria showed 100% susceptibility to meropenem, including both non-lactose fermenters and the Enterobacterales group. *Pseudomonas aeruginosa* exhibited the highest antimicrobial resistance rates against penicillins (Piperacillin + Tazobactam (PIT)), cephalosporins (Cefepime (CPM), Ceftazidime (CAZ), and Cefoxitin (CFO)), monobactams (Aztreonam (ATM)), and quinolones (Ciprofloxacin (CIP) and Levofloxacin (LEV)). The Enterobacterales group also showed full susceptibility (100%) to PIT but demonstrated higher resistance rates to Amoxicillin + Clavulanic Acid (AMC), quinolones, and Sulfamethoxazole + Trimethoprim (SUT).

*Staphylococcus* spp. (*n* = 30) exhibited 100% susceptibility to vancomycin and 97% (*n* = 29) to linezolid, with the highest resistance observed to macrolides (Erythromycin (ER)) and tetracyclines (TET). Coagulase-negative staphylococci (CoNS) displayed a higher resistance profile than *S. aureus*, particularly to cefoxitin, quinolones, clindamycin (CLIN), and SUT. Among other Gram-positive bacteria, such as *Enterococcus faecalis*, 50% (*n* = 3) showed resistance to quinolones and one isolate (17%) was resistant to vancomycin. All data are summarized in Table 3.

Table 4 presents the multidrug resistance patterns among the main identified isolates. *P. aeruginosa* showed the highest prevalence of MDR (48%) and PDR (29%) strains. Among Enterobacteriaceae, *P. mirabilis* and *E. coli* exhibited greater susceptibility, although a portion displayed MDR behavior (34% and 40%, respectively). Within *Staphylococcus* spp., *S. aureus* demonstrated an MDR-compatible profile (47%), while PDR phenotypes were observed in *S. epidermidis* (20%) and in the CoNS group (13%).

The associations between clinical-demographic variables and microbiological outcomes are summarized in Table 5. Most clinical variables, including gender (*p* = 0.394), age (*p* = 1.000), diabetes mellitus (*p* = 0.651), hypertension (*p* = 0.202), and leprosy (*p* = 0.358), showed no significant association with the detection of MDR pathogens. Similarly, the duration of the ulcer, stratified into 10 years (*p* = 0.141), 11–20 years (*p* = 1.000), and >20 years (*p* = 0.113, did not correlate with increased resistance profiles. Notably, a significant association was found between the sampling method and the identification of MDR strains, as detailed in the comprehensive statistical analysis. Biopsy samples were significantly more likely to yield MDR isolates compared to swabs (*p* = 0.008). No significant correlation was observed between the isolation of *P. aeruginosa* and XDR profiles (*p* = 0.115).

In the phenotypic evaluation of ESBL production, 19% (*n* = 8) positivity was observed among isolates belonging to the order Enterobacterales, represented by *P. mirabilis* (24%), *P. stuartii* (100%), *P. rettgeri* (33%), and *E. coli* (17%). Genotypic analysis revealed a higher prevalence of the *blaTEM* (36%) and *blaSHV* (36%) genes compared to *blaCTX-M1* (27%). Although not phenotypically detected, 67% (*n* = 2) of *K. oxytoca* isolates were positive for *blaTEM*, and 33% (*n* = 1) exhibited the coexistence of all three genes.

Regarding *ampC* production in *Enterobacterales*, 17% (*n* = 7) of isolates showed phenotypic expression, which was genotypically confirmed by the presence of the *blaCMY-2* gene in 56% (*n* = 23). Phenotypically, only one *P. mirabilis* isolate exhibited *ampC* production, while genotypic detection was present in 43% (*n* = 9). A similar pattern was observed for *E. coli*, with 83% (*n* = 5) molecular positivity for *blaCMY-2*, despite the absence of phenotypic expression. All results are summarized in Table 6.

The genus *Staphylococcus* spp. exhibited 33% of isolates with phenotypic resistance to oxacillin. Molecular detection revealed that 40% (*n* = 12) of microorganisms carried the gene *mecA*. The susceptibility of *S. aureus* to oxacillin remained consistent among isolates, with higher resistance observed in CoNS. In *S. aureus*, 20% (*n* = 3) showed phenotypic resistance to cefoxitin, confirmed by 33% (*n* = 5) *mecA* positive isolates, suggesting MRSA strains. Among CoNS, *S. epidermidis*, *S. cohnii*, *S. lugdunensis*, *S. pettenkoferi*, and *S. haemolyticus* demonstrated concomitance between the OXA-resistant phenotype and *mecA* positivity (40–100%).

MLSb-type resistance was detected in half (*n* = 15) of the *Staphylococcus* spp. isolates, with higher activity in *S. cohnii* (100%), *S. argenteus* (100%), *S. hominis* (100%), and *S. epidermidis* (80%), and a predominance of the constitutive cMLSb phenotype (30%). In *Staphylococcus* spp., both *ermA* and *ermC* genes were detected in 37% and 40% of isolates, respectively, with a slight predominance of *ermA*. The coexistence of *ermA* and *ermC* was observed in only two isolates (7%) from the CoNS group, according to the data in Table 7.

Among the 33% (*n* = 10) of *Staphylococcus* spp. isolates exhibiting phenotypic resistance to oxacillin, 50% (*n* = 5) concomitantly carried the MLSb phenotype, with a predominance (*n* = 4) of the cMLSb profile (40%). Sixty-seven percent (*n* = 2) of oxacillin-resistant *S. aureus* (MRSA) isolates exhibited cMLSb resistance, while CoNS showed 43% (*n* = 3) of the MLSb phenotype, with 29% (*n* = 2) in its constitutive form. The same pattern observed phenotypically was reflected in the detection of the *mecA* gene in *Staphylococcus* spp. (33%), with 60% (*n* = 6) of *mecA* positive isolates carrying at least one gene from the *erm* complex, among which the CoNS group exhibited higher positivity (67%) compared to *S. aureus* (60%). All results are summarized in Table 8.

## 4. Discussion

The clinical profile observed in this study reflects the complex nature of chronic wound healing, where aging, male predominance, and comorbidities such as vascular and neurological disorders act as key drivers of chronicity [1,2,3]. Interestingly, the detection of MDR pathogens was not significantly associated with demographic variables such as gender (*p* = 0.394) or age (*p* = 1.000), nor with comorbidities including diabetes mellitus (*p* = 0.651) or leprosy (*p* = 0.358). These findings suggest that, in this specialized setting, MDR colonization represents a widespread risk across the patient population, regardless of individual clinical characteristics. Furthermore, the trend toward longer-standing lesions in colonized patients, despite the lack of statistical significance for *Staphylococcus* spp. (*p* = 0.274), reinforces the role of microbial persistence in recalcitrant ulcers.

The microbial landscape was dominated by *P. aeruginosa*, *P. mirabilis*, and *S. aureus*, consistent with global literature [24,25]. In addition to this overall distribution, stratified analyses suggest that host-related factors may influence colonization patterns. For instance, 86.7% of hypertensive patients carried *P. aeruginosa* (*p* = 0.056), indicating that hypertension may contribute to a physiological niche favoring this species [25].

In a study conducted by Garcia et al. [26], which characterized bacterial colonization and microbial load in leg ulcers, an association between *P. mirabilis* and infected ulcers was reported, corroborating our findings. Additionally, *P. mirabilis* and coagulase-negative *Staphylococcus* (CoNS) were significantly associated with female patients (*p* = 0.038), suggesting a possible influence of host-related ecological factors. The statistical significance of these associations highlights patterns that warrant further investigation in larger cohorts. Moreover, although *P. aeruginosa* exhibited high resistance rates, no significant correlation was observed between its isolation and XDR profiles (*p* = 0.115), indicating that, in this cohort, resistance may be substantial but not maximal.

A key finding of this study relates to the sampling methodology. A significant association was identified between the sampling technique and the recovery of multidrug-resistant strains (*p* = 0.008), with biopsy samples showing a higher probability of isolating these organisms compared to superficial swabs. This supports the hypothesis that resistant pathogens and biofilms are predominantly located in deeper layers of the ulcer bed, where they are less affected by topical treatments and surface cleansing. These results highlight the clinical importance of deep tissue sampling for accurate microbiological diagnosis in chronic wounds [27].

Most antimicrobial susceptibility rates in *Staphylococcus* spp. fall within the expected range for community-acquired strains. However, the indiscriminate use of antibiotics during the SARS-CoV-2 pandemic may have contributed to the emergence of resistant phenotypes, particularly affecting susceptibility to erythromycin derivatives [28]. The 100% susceptibility to vancomycin observed across the genus is epidemiologically relevant, as this antimicrobial remains a cornerstone for the treatment of Gram-positive infections following the emergence of oxacillin resistance. In this context, the detection of the *mecA* gene remains essential for monitoring β-lactam resistance and tracking MRSA dissemination.

Lincosamides may represent an alternative therapeutic option for oxacillin-resistant strains. However, phenotypic detection of MLSb-type resistance varies according to regional characteristics, prior antimicrobial exposure, and local resistance patterns. At the molecular level, the prevalence of *ermA* may range from 10% to 60%, whereas *ermC* is generally less frequent, reaching up to 30%. In a review by Assefa et al. [28], 26.8% of *S. aureus mecA*-positive isolates also exhibited MLSb production, primarily involving *erm* and *msrA* genes. These findings align with our results, in which 50% (*n* = 5) of oxacillin-resistant *Staphylococcus* spp. isolates exhibited the MLSb phenotype, and 60% of *mecA*-positive strains carried at least one *erm* gene, reinforcing the potential limitations of clindamycin therapy in MRSA infections.

The high resistance profile observed in *P. aeruginosa* to piperacillin–tazobactam, cephalosporins, and monobactams may be attributed to its genomic complexity, which enables the expression of diverse regulatory mechanisms that enhance metabolic adaptability. Although this species demonstrated elevated resistance rates, this did not translate into a significant association with XDR profiles, suggesting partial rather than extreme resistance in this cohort. Intrinsic resistance mechanisms, including efflux pumps and the production of inactivating enzymes such as AmpC, likely contribute to these patterns, particularly regarding reduced susceptibility to fluoroquinolones and β-lactams [29]. Although 77% (*n* = 27) of isolates exhibited phenotypic positivity for AmpC, molecular confirmation was not performed, given the intrinsic nature of its expression in this species.

Within the order Enterobacterales, antimicrobial susceptibility patterns were consistent with those typically observed in community settings, with higher resistance rates to amoxicillin–clavulanate (42%), ciprofloxacin (45%), levofloxacin (37%), and sulfamethoxazole–trimethoprim (39%). ESBL production was phenotypically detected in 19% (*n* = 8) of isolates and confirmed by the presence of genes such as *blaCTX-M-1*. Although *blaTEM-1* and *blaSHV-1* are not classified as ESBLs, they encode narrow-spectrum β-lactamases and may contribute synergistically to antimicrobial resistance. Furthermore, point mutations in these genes can give rise to ESBL variants such as *blaTEM-52* and *blaSHV-12*.

*E. coli* isolates showed a higher frequency of resistance genes compared to phenotypic expression, suggesting variable gene expression or regulation. Although expression analyses such as RT-PCR were not performed, this discrepancy indicates a potential latent resistance reservoir not fully captured by standard phenotypic methods. In Enterobacterales, phenotypic AmpC production was observed in 17% (*n* = 7) of isolates, a lower prevalence compared to ESBLs. Unlike *P. aeruginosa*, where AmpC expression is intrinsic, in Enterobacterales it may be inducible or plasmid-mediated. This dynamic regulation has important clinical implications, as exposure to β-lactam antibiotics may select for AmpC-producing mutants. Genes such as *blaCMY-2* play a key role in this process, and their plasmid-mediated dissemination facilitates spread. The absence of phenotypic expression in some genotypically positive isolates may reflect regulatory mechanisms such as induction thresholds or chromosomal derepression, potentially influencing antimicrobial response under clinical conditions [30].

The challenges in managing infectious conditions in chronic wounds are closely linked to antimicrobial resistance, exacerbated by the widespread and often indiscriminate use of antibiotics. The detection of clinically relevant resistance genes—including *mecA*, *blaTEM*, *blaSHV*, *blaCTX-M1*, *ermA*, *ermC*, and *blaCMY-2*—confirms the presence of multidrug resistance among bacteria isolated from chronic ulcers. However, distinguishing between colonization and true infection remains essential to guide appropriate therapy. Therefore, antimicrobial use should be guided by clinical evidence, microbiological findings, and stewardship strategies aimed at preserving the effectiveness of available treatments [31].

### Limitations of the Study

Despite the clinical relevance of our findings, this study has some limitations. First, the research was conducted at a single specialized dermatological center with a specific diagnostic demand, which may limit the generalizability of the results to general hospitals or other geographical regions. Additionally, the sample size was determined by convenience and did not involve prior power calculations, which warrants caution when generalizing the prevalence rates and statistical associations to larger populations. Furthermore, the identification of Gram-negative bacteria was based on biochemical and semi-automated methods rather than proteomic techniques like MALDI-TOF. However, we mitigated these limitations by implementing rigorous monthly quality control with ATCC reference strains and employing molecular identification (ITS-PCR and sequencing) for Gram-positive isolates to ensure taxonomic precision. Future multicenter studies with larger cohorts and a broader range of molecular resistance markers are needed to further elucidate the complex epidemiology of chronic wound infections in Southeastern Brazil.

## 5. Conclusions

This study demonstrates a high burden of polymicrobial colonization in chronic ulcers, with a predominance of Gram-negative bacilli, particularly *P. aeruginosa* and *P. mirabilis*, as well as species of the genus Staphylococcus. The presence of MDR and PDR phenotypes, in association with clinical and socioeconomic factors, underscores the complexity of these lesions. Notably, the sampling methodology played a decisive role in these findings, as isolates recovered via biopsy were significantly more likely to reveal MDR pathogens compared to superficial swabs (*p* = 0.008), highlighting the presence of resistant strains in deeper tissue layers. The detection of genetic determinants of resistance, including *blaTEM*, *blaSHV*, *blaCMY-2*, *mecA*, *ermA*, and *ermC*, reveal discrepancies between genotypic and phenotypic profiles, suggesting the presence of unexpressed resistance that may be inducible during treatment. The high prevalence of *P. aeruginosa*, coupled with comorbidities such as hypertension, points to its potential adaptation to vascularly compromised microenvironments. Although vancomycin and meropenem retain in vitro activity, the high frequency of *mecA*-positive strains and the MLSB phenotype as well as the phenotypic expression of AmpC in P. aeruginosa limit therapeutic options and complicate empirical antimicrobial selection. In this context, effective management of these lesions would benefit from a transition from empirical approaches to strategies guided by local molecular epidemiology. Continuous surveillance of resistance markers is essential to optimize wound healing, curb the dissemination of resistant pathogens, and preserve the efficacy of the antimicrobial arsenal.

## Figures and Tables

**Table 1:** Demographic and clinical characteristics of the study population.

	N (%)	Mean + SD		N (%)
Age (years)	-	63.8 ± 11.6	Dressing change	
			Once daily	22 (67%)
Male	21 (64%)		Only at hospital	5 (15%)
Female	12 (36%)		Twice daily	3 (9%)
			Every other day	2 (6%)
Education		±5.04	Twice a week	1 (3%)
Incomplete	20 (61%)			
Basic education	5 (15%)		Wound etiology	
Secondary education	6 (18%)		Venous	30 (91%)
Higher Education	2 (6%)		Other	3 (9%)
				
Occupation			Comorbidities *	
Retired	16 (48%)		Arterial hypertension	18 (55%)
Homemaker	5 (15%)		Leprosy	9 (27%)
Other	12 (37%)		Circulatory diseases	8 (24%)
			Diabetes Mellitus	4 (12%)
Wound duration (years)			Others	11 (33%)
≤10 years	19 (58%)	14.5 ± 14.4		
11–20 years	5 (15%)			
>20 years	9 (27%)			
				
Number of wounds				
One wound	14 (42.5%)	1.87 ± 1.08		
Two wounds	14 (42.5%)			
Three wounds	2 (6%)			
Four wounds	1 (3%)			
Five wounds	2 (6%)			
				
BMI (Body Mass Index)		27.56 ± 5.04		

Results are expressed as mean ± SD or as N (%). BMI: body mass index. * Patients may present more than one comorbidity.

**Table 2:** Distribution of bacterial species detected by sampling method: Swab only, Biopsy only, and both combined. Total counts and percentages are provided.

	Swab	Biopsy	Swab + Biopsy	Total
*P. aeruginosa*	16	7	8	31 (27%)
*P. mirabilis*	8	9	4	21 (18%)
*S. aureus*	5	5	5	15 (13%)
*E. coli*	4	0	2	6 (5%)
*E. faecalis*	3	2	1	6 (5%)
*S. epidermidis*	3	1	1	5 (4%)
*K. oxytoca*	3	0	0	3 (3%)
*P. rettgeri*	0	3	0	3 (3%)
*Acinetobacter* spp.	2	0	1	3 (3%)
*S. saprophyticus*	2	0	0	2 (2%)
*S. cohnii*	2	0	0	2 (2%)
*M. morganii*	1	0	1	2 (2%)
*Cronobacter sakazakii*	0	2	0	2 (2%)
*Coryneobacterium* spp.	0	0	2	2 (2%)
*S. lugdunensis*	1	1	0	2 (2%)
*Pseudomonas luteola*	0	0	1	1 (1%)
*Providencia stuartii*	0	0	1	1 (1%)
*P. vulgaris*	0	1	0	1 (1%)
*S. pettenkoferi*	1	0	0	1 (1%)
*S. argenteus*	1	0	0	1 (1%)
*S. dysgalactiae*	0	1	0	1 (1%)
*S. haemolyticus*	0	1	0	1 (1%)
*Y. intermedia*	0	1	0	1 (1%)
*K. ascorbata*	0	1	0	1 (1%)
*K. pneumoniae*	0	0	1	1 (1%)
*S. hominis*	0	1	0	1 (1%)
Total	52 (100%)	36 (100%)	28 (100%)	116 (100%)

**Table 3:** Antimicrobial Resistance Profile of the Major Isolates Identified.

	AMC	PIT	CFO	CPM	CAZ	ATM	IPM	MPM	CIP	LEV	VAN	ERI	CLIN	TET	LNZ	RIF	SUT	AMI	TOB
*P. aeruginosa*	-	20 (65%)	-	24 (77%)	24 (77%)	24 (77%)	8 (26%)	0	14 (45%)	15 (48%)	-	-	-	-	-	-	-	4 (13%)	9 (29%)
Enterobacterales	16 (42%)	0	4 (11%)	4 (11%)	8 (21%)	3 (8%)	2 (5%)	0	17 (45%)	14 (37%)	-	-	-	-	-	-	15 (39%)	2 (5%)	-
*P. mirabilis*	6 (29%)	0	1 (5%)	3 (14%)	5 (24%)	1 (5%)	1 (5%)	0	7 (33%)	4 (19%)	-	-	-	-	-	-	6 (29%)	0	-
*E. coli*	1 (17%)	0	1 (17%)	1 (17%)	1 (17%)	1 (17%)	1 (17%)	0	4 (67%)	4 (67%)	-	-	-	-	-	-	3 (50%)	0	-
*S. aureus*	-	-	3 (20%)	-	-	-	-	-	4 (27%)	4 (27%)	0	9 (60%)	6 (40%)	7 (47%)	1 (7%)	1 (7%)	4 (27%)	-	-
CoNS			7 (47%)	-	-	-	-	-	9 (60%)	9 (60%)	0	9 (60%)	10 (67%)	13 (87%)	0	4 (27%)	9 (60%)	-	-
*S. epidermidis*	-	-	2 (40%)	-	-	-	-	-	3 (60%)	3 (60%)	0	4 (80%)	4 (80%)	4 (80%)	0	1 (20%)	4 (80%)	-	-
*E. faecalis*	-	1 (17%)	-	-	-	-	0	-	3 (50%)	3 (50%)	1 (17%)	-	-	-	1 (17%)	-	-	-	-

AMC: Amoxicillin + Clavulanic Acid; PIT: Piperacillin + Tazobactan; CFO: Cefoxitin; CPM: Cefepime; CAZ: Ceftazidime; ATM: Aztreonam; IMP: Imipenem; MPM: Meropenem; CIP: Ciprofloxacin; LEV: Levofloxacin; VAN: Vancomycin; ERI: Erythromycin; CLIN: Clindamycin; TET: Tetracycline; LNZ: Linezolid; SUT: Sulfamethoxazole + Trimethoprim; AMI: Amikacin and TOB: Tobramycin. CoNS: Coagulase-negative staphylococci.

**Table 4:** Multidrug Resistance Patterns (R0–R6) of the Main Bacterial Isolates.

	R0	R1	R2	R3	R4	R5	R6
*P. aeruginosa*	0	4 (13%)	3 (10%)	10 (32%)	5 (16%)	9 (29%)	0
Enterobacterales	14 (34%)	4 (10%)	9 (22%)	11 (27%)	3 (7%)	0	0
*P. mirabilis*	8 (38%)	3 (10%)	4 (19%)	5 (24%)	1 (5%)	0	0
*E. coli*	2 (33%)	0	2 (33%)	1 (17%)	1 (17%)	0	0
*S. aureus*	3 (20%)	1 (7%)	4 (27%)	4 (27%)	2 (13%)	0	1 (7%)
*ECN*	0	0	2 (13%)	4 (27%)	3 (20%)	4 (27%)	2 (13%)
*S. epidermidis*	0	0	0	0	1 (20%)	3 (60%)	1 (20%)
*E. faecalis*	2 (33%)	1 (17%)	2 (33%)	1 (17%)	0	0	0

R0: no resistance, R1: resistance to 1 drug class, R2: resistance to 2 drug classes, R3: resistance to 3 drug classes, R4: resistance to 4 drug classes, R5: resistance to 5 drug classes, R6: resistance to 6 drug classes.

**Table 5:** Statistical analysis of clinical and methodological variables performed.

Variable Category	Comparison/Correlation	*p*-Value *
Demographics	Gender vs. MDR	0.394
	Gender vs. *P. mirabilis*	0.038 *
	Gender vs. CoNS Group	0.038 *
	Age vs. MDR	1.000
Comorbidities	Diabetes Mellitus vs. MDR	0.651
	Hypertension vs. MDR	0.202
	Hypertension vs. *P. aeruginosa*	0.056
	Leprosy vs. MDR	0.358
Ulcer Characteristics	Duration (10 years) vs. MDR	0.141
	Duration (11–20 years) vs. MDR	1.000
	Duration (>20 years) vs. MDR	0.113
	Duration vs. *Staphylococcus* spp.	0.274
Methodology	Swab vs. MDR	0.212
	Biopsy vs. MDR	0.008 *
Microbiology	*P. aeruginosa* vs. XDR	0.115

* Statistical significance determined by Fisher’s Exact Test or Pearson’s Chi-square test (*p* < 0.05).

**Table 6:** Phenotypic and Genotypic Detection of β-Lactamase Production Among the Main Bacterial Isolates.

	Total	ESBL	*blaTEM*	*blaSHV*	*blaCTX-M1*	*blaTEM + blaSHV*	*blaTEM + blaCTX-M1*	*blaSHV+ blaCTX-M1*	*blaTEM + blaSHV + blaCTX-M1*	ampC	*blaCMY-2*
Enterobacterales	41	8 (19%)	15 (36%)	15 (36%)	11 (27%)	4 (10%)	1 (2%)	2 (5%)	2 (5%)	7 (17%)	23 (56%)
*P. mirabilis*	21	5 (24%)	7 (33%)	3 (14%)	4 (19%)	0	1 (5%)	0	0	1 (5%)	9 (43%)
*E. coli*	6	1 (17%)	4 (67%)	6 (100%)	2 (33%)	3 (50%)	0	1 (17%)	1 (17%)	0	5 (83%)
*P. rettgeri*	3	1 (33%)	1 (33%)	1 (33%)	1 (33%)	0	0	0	0	1 (33%)	1 (33%)
*K. oxytoca*	3	0	2 (67%)	1 (33%)	1 (33%)	0	0	0	1 (33%)	0	2 (67%)
*M. morganii*	2	0	0	0	1 (50%)	0	0	0	0	2 (100%)	2 (100%)
*C. sakazakii*	2	0	0	1 (50%)	0	0	0	0	0	1 (50%)	1 (50%)
*P. stuartii*	1	1 (100%)	1 (100%)	1 (100%)	0	1 (100%)	0	0	0	1 (100%)	1 (100%)
*Y. intermedia*	1	0	0	1 (100%)	1 (100%)	0	0	1 (100%)	0	1 (100%)	1 (100%)
*P. vulgaris*	1	0	0	0	1 (100%)	0	0	0	0	0	1 (100%)
*K. pneumoniae*	1	0	0	1 (100%)	0	0	0	0	0	0	0
*P. aeruginosa*	31	-	-	-	-	-	-	-	-	27 (77%)	**-**

Phenotypic (ESBL and AmpC) and genotypic (*blaTEM*, *blaSHV*, *blaCTX-M1*, *blaCMY-2*) detection of β-lactamase production among the main bacterial isolates. Data are presented as number and percentage of positive isolates.

**Table 7:** Distribution of resistance genes and phenotypic profiles related to oxacillin and clindamycin resistance in *Staphylococcus* spp. Isolates.

	Total	OXA	*mecA*	cMLSb	iMLSb	MLSb	*ermA*	*ermC*	*ermAC*
*Staphylococcus* spp.	30	10 (33%)	12 (40%)	9 (30%)	6 (20%)	15 (50%)	12 (40%)	11 (37%)	2 (7%)
*S. aureus*	15	3 (20%)	5 (33%)	4 (27%)	2 (13%)	6 (40%)	5 (33%)	5 (33%)	0
*S. epidermidis*	5	2 (40%)	3 (60%)	2 (40%)	2 (40%)	4 (80%)	2 (40%)	3 (60%)	0
*S. saprophyticus*	2	1 (50%)	0	0	0	0	0	0	0
*S. cohnii*	2	1 (50%)	1 (50%)	2 (100%)	0	2 (100%)	2 (100%)	0	0
*S. lugdunensis*	2	1 (50%)	1 (50%)	0	1 (50%)	1 (50%)	1 (50%)	0	0
*S. pettenkoferi*	1	1 (100%)	1 (100%)	0	0	0	1 (100%)	1 (100%)	1 (100%)
*S. argenteus*	1	0	0	1 (100%)	0	1 (100%)	0	1 (100%)	0
*S. haemolyticus*	1	1 (100%)	1 (100%)	0	0	0	0	0	0
*S. hominis*	1	0	0	0	1 (100%)	1 (100%)	1 (100%)	1 (100%)	1 (100%)

OXA: Phenotypic resistance to oxacillin; *mecA*: Gene encoding methicillin resistance; cMLSb: Constitutive resistance phenotype to macrolide-lincosamide-streptogramin B (MLSb) antibiotics; iMLSb: Inducible resistance phenotype to macrolide-lincosamide-streptogramin B (MLSb) antibiotics; MLSb: Total MLSB phenotype (cMLSb + iMLSb); *ermA*, *ermC*, *ermAC*: Genes encoding resistance to MLSb antibiotics.

**Table 8:** Coexistence of Oxacillin and Clindamycin Resistance in *Staphylococcus* spp.

	Total	OXA	OXA + iMLSb	OXA + cMLSb	OXA + MLSb
*Staphylococcus* spp.	30	10 (33%)	1 (10%)	4 (40%)	5 (50%)
*S. aureus*	15	3 (20%)	0	2 (67%)	2 (67%)
SCN	15	7 (47%)	1 (14%)	2 (29%)	3 (43%)
*S. epidermidis*	5	2 (40%)	1 (20%)	1 (20%)	2 (40%)
					
	Total	*mecA* +	*mecA* + *ermA*	*mecA* + *ermC*	*mecA* + *erm*
*Staphylococcus* spp.	30	10 (33%)	4 (40%)	2 (20%)	6 (60%)
*S. aureus*	15	4 (27%)	1 (25%)	1 (25%)	2 (50%)
SCN	15	6 (40%)	3 (50%)	1 (17%)	4 (67%)
*S. epidermidis*	5	3 (60%)	2 (40%)	1 (20%)	3 (60%)

Phenotypic and genotypic detection of oxacillin and clindamycin resistance in *Staphylococcus* isolates from chronic lower limb ulcers. Data are expressed as number and percentage of isolates.

## Data Availability

The original contributions presented in this study are included in the article. Further inquiries can be directed to the corresponding author.

## Abbreviations

The following abbreviations are used in this manuscript:

AMC	Amoxicillin + Clavulanic Acid
AMP	AmpC β-lactamase
ANVISA	Brazilian Health Regulatory Agency
ATM	Aztreonam
BHI	Brain Heart Infusion
BMI	Body Mass Index
CAZ	Ceftazidime
CFO	Cefoxitin
CIP	Ciprofloxacin
CLIN	Clindamycin
CLSI	Clinical and Laboratory Standards Institute
CoNS	Coagulase-negative *Staphylococcus*
CPM	Cefepime
cMLSb	Constitutive macrolide-lincosamide-streptogramin B resistance phenotype
DNA	Deoxyribonucleic acid
ERI	Erythromycin
ESBL	Extended-spectrum β-lactamase
IPM	Imipenem
iMLSb	Inducible macrolide-lincosamide-streptogramin B resistance phenotype
ITS-PCR	Internal transcribed spacer polymerase chain reaction
LEV	Levofloxacin
LNZ	Linezolid
MDR	Multidrug-resistant
MIC	Minimum inhibitory concentration
MLSb	Macrolide-lincosamide-streptogramin B resistance phenotype
MPM	Meropenem
MRSA	Methicillin-resistant *Staphylococcus aureus*
OXA	Oxacillin
PCR	Polymerase chain reaction
PDR	Pan-drug-resistant
PIT	Piperacillin + Tazobactam
RIF	Rifampicin
SD	Standard deviation
SUT	Sulfamethoxazole + Trimethoprim
TET	Tetracycline
TOB	Tobramycin
VAN	Vancomycin
XDR	Extensively drug-resistant
